# Motor Cortical Gamma Oscillations: What Have We Learnt and Where Are We Headed?

**DOI:** 10.1007/s40473-018-0151-z

**Published:** 2018-04-27

**Authors:** Magdalena Nowak, Catharina Zich, Charlotte J. Stagg

**Affiliations:** 1 Wellcome Centre for Integrative Neuroimaging, FMRIB, Nuffield Department of Clinical Neurosciences, University of Oxford, Oxford, OX3 9DU UK; 2 Oxford Centre for Human Brain Activity, Wellcome Centre for Integrative Neuroimaging, Department of Psychiatry, University of Oxford, Oxford, OX3 7JX UK

**Keywords:** Gamma oscillations, Motor cortex, Motor control, Movement, Motor learning

## Abstract

**Purpose of Review:**

An increase in oscillatory activity in the *γ*-frequency band (approximately 50–100 Hz) has long been noted during human movement. However, its functional role has been difficult to elucidate. The advent of novel techniques, particularly transcranial alternating current stimulation (tACS), has dramatically increased our ability to study *γ* oscillations. Here, we review our current understanding of the role of *γ* oscillations in the human motor cortex, with reference to *γ* activity outside the motor system, and evidence from animal models.

**Recent Findings:**

Evidence for the neurophysiological basis of human *γ* oscillations is beginning to emerge. Multimodal studies, essential given the necessarily indirect measurements acquired in humans, are beginning to provide convergent evidence for the role of *γ* oscillations in movement, and their relationship to plasticity.

**Summary:**

Human motor cortical *γ* oscillations appear to play a key role in movement, and relate to learning. However, there are still major questions to be answered about their physiological basis and precise role in human plasticity. It is to be hoped that future research will take advantage of recent technical advances and the physiological basis and functional significance of this intriguing and important brain rhythm will be fully elucidated.

## Introduction

Motor cortical gamma-frequency oscillations (*γ*; 30–100 Hz) have been recorded from multiple brain regions, across many spatial scales, including extracellular recordings within the brain (single- or multi-unit, local field potentials [LFP]) [1, 2, 3•], surface electrocorticographic (ECoG) recordings [4, 5], and scalp-recorded EEG/MEG in humans [6–8]. A substantial body of research performed in hippocampal slice preparations, as well as in neocortical and other brain regions suggests that *γ* oscillations arise from interactions between reciprocally connected inhibitory interneurons and pyramidal cells [9]. While motor cortex *γ* activity is considered to be prokinetic in nature [7, 10•, 11], this view does not fully reflect its functional complexity [12, 13], and its precise role in motor processes remains to be elucidated. Recently developed interventional approaches, such as optogenetic tools in animals and transcranial alternating current stimulation (tACS) in humans, offer unique opportunity to study mechanisms underlying rhythmic activity and establish a causal relationship between motor cortical oscillations and various aspects of motor control.

This review aims to briefly summarise the mechanisms underlying *γ* oscillations, focusing on the primary motor cortex (M1). In addition, the functional significance of neocortical motor *γ* activity and the current state of the field are discussed. Finally, the contribution and limitations, of optogenetic- and tACS-based approaches to inform our understanding of motor cortical *γ* activity are reviewed.

## Mechanisms of Neocortical *γ* Oscillations

Identifying the mechanisms underlying neocortical *γ* oscillations is vital for understanding their precise role in motor processes as well as for the development and assessment of therapeutic interventions aimed at modulating motor γ activity.

*γ* oscillations are considered to emerge through activation of reciprocally connected excitatory pyramidal neurons and inhibitory interneurons (with increasing involvement of interneuron-only networks in a higher frequency *γ* activity), whose period is regulated by the decay time constant of GABA_A_-mediated synaptic current [9, 14]. Among the various interneuron subtypes, fast-spiking (FS) parvalbumin positive (PV) cells are a key element of neocortical *γ* oscillations [15, 16], displaying unique electrophysiological properties, such as intrinsic resonance in the *γ*-frequency band [17, 18]. A recent pharmacological dissection of *γ* oscillations in rat M1 in vitro has shown a reduction of *γ* oscillatory power by the GABA_A_ and AMPA receptor antagonists; an increase in the *γ* power as a result of an augmentation of GABA_A_ receptor activity or the blockade of GABA_B_, NMDA and metabotropic glutamate receptor activity; and a relatively complex effect of the above pharmacological agents on the *γ* frequency [3•]. Another important ingredient of *γ* oscillations are electrical synapses, both between pyramidal cells and between interneurons [19]. The blockade of gap junctions has been shown to abolish *γ* activity in M1 in vitro [3•].

While the modelling and animal studies have played a vital role in elucidating the physiological mechanisms underpinning *γ* activity, it is not clear whether these findings can be directly translated into humans. To date, only a limited number of human studies has been performed, few of which have focused on the motor domain. The results of these have often been inconclusive, or contradictory, possibly due to the inherent difficulties in recording a low-signal oscillation with necessarily indirect, non-invasive, noisy methods.

Neither diazepam, a non-specific GABA_A_ receptor modulator, nor tiagabine, a GABA transporter 1 (GAT-1) blocker, modulate movement-related *γ* synchronisation (MRGS) power or frequency in M1, as measured using MEG, suggesting that MRGS may not be a GABA_A_-mediated process, in contradiction to the animal literature [20, 21]. However, MRGS frequency, but not power, has been found to be related to M1 GABA concentration, as measured using magnetic resonance spectroscopy (MRS) [22]. Further, driving cortical *γ* oscillations has been demonstrated to reduce transcranial magnetic stimulation (TMS)-assessed synaptic GABA_A_ inhibition in the human M1 [23•]. While the above findings may appear to be inconsistent, it is worth noting that the techniques employed to measure GABA activity may reflect different aspects of GABAergic signalling. For example, the TMS protocol employed in our recent study [23•] measures primarily synaptic GABA_A_ activity [24], whereas MRS is likely to reflect extra-synaptic, tonic, inhibition [25, 26], although further investigation may be required [27]. Moreover, MEG-detected MRGS appears to be stronger in earlier trials in a sequence (which may influence MRGS over extended trials) [7], and cannot be reliably detected in all individuals [23•], thus adding additional challenge to studying this type of oscillatory activity in humans.

It is important to note, particularly when considering systems-level activity, that *γ* oscillations are routinely coupled to oscillatory activity at other frequencies, most commonly at the theta (θ) frequency [3•, 9, 28]. Outside the motor system, it has long been hypothesised that this cross-frequency coupling enables local *γ*-generating networks to be coupled by long-range connections [29]. In the motor domain, M1 *γ* activity has been shown to be coherent with that in the subcortical basal ganglia structures, with evidence that cortical *γ* may be driven by subcortical *γ* [12, 30]. In addition, computational modelling approaches have provided evidence that different mechanisms may be responsible for local pyramidal-interneuronal *γ* as opposed to *γ* driven by basal ganglia-thalamocortical sources [31], although it remains to be examined how these small- and large-scale *γ* networks interact.

## Movement-Related Changes in *γ* Amplitude and Their Function

Movement-related changes in *γ* amplitude in the human M1 were first observed in ECoG recordings [5], and have subsequently been observed using a variety of invasive [4, 32–34] and non-invasive [35–38] recording techniques. Specifically, an increase in the amplitude of *γ* oscillations occurs during movement execution, a process referred to as movement-related *γ* synchronisation (MRGS) [5, 7, 35, 39]. The MRGS has been described as spatially and temporally more specific, i.e., it shows a greater somatotopic organisation and lateralisation as well as a greater temporal congruence with the movement than concurrently occurring movement-related changes in *α*- and *β*-frequency oscillations [7, 40]. However, in contrast to *α* and *β* oscillations, the majority of studies have reported no changes in *γ* oscillations prior to movement onset [7, 8]. For these reasons, it has been suggested that movement-related changes in *γ* amplitude could be the result of afferent proprioceptive feedback to the motor cortex during movement [7, 41]. Others have argued that the lack of MRGS during both passive movements and movement observation indicates that movement-related changes in *γ* oscillations relate to more active motor control processes rather than just to afferent proprioceptive feedback [7]. A parsimonious explanation for these findings might be that movement-related changes in *γ* oscillations stemming from sensorimotor areas are involved in the processing of afferent proprioceptive feedback in order to control movements. However, a recent study demonstrated movement-related changes in *γ* oscillations in the absence of proprioceptive feedback signalling using the mirror box paradigm [42], suggesting that the hypothesis of *γ* oscillations solely reflecting proprioception may be too simplistic.

Beyond cortical changes in movement-related *γ* oscillations, direct recordings from patients with Parkinson’s disease and animal models have demonstrated that the degree of local, subcortical MRGS is related to velocity, effort, or force levels underpinning movements [43–45]. The importance of subcortical *γ* is underlined by the converging evidence that supressed *γ* oscillatory activity (and enhanced *β* oscillatory activity) in the cortico-basal ganglia loop circuits is directly linked to symptoms in patients with Parkinson’s disease [46–50]. Moreover, it has been demonstrated that effective pharmacologic treatments (i.e., levodopa and dopamine receptor agonists) enhance subcortical *γ* and reduce subcortical *β* oscillatory activity [51, 52]. The opposing direction of changes in *γ* and *β* oscillatory activity in Parkinson’s disease suggests a reciprocal relationship between these two frequency ranges. Further, evidence supporting this hypothesis was provided by a recent study demonstrating reciprocity of *γ* and *β* oscillatory activity in the supplementary motor area (SMA) and pre-SMA of monkeys during performance of a bimanual motor sequence [53•].

Taken together, these studies suggest that movement execution is related to an increase in *γ* amplitude in cortical and subcortical regions and, further, that the degree of that increase is closely related to movement properties, both in health and disease.

## The Role of *γ* Oscillations in Plasticity

Outside the motor system, *γ* oscillations have routinely been suggested to reflect local synaptic plasticity. *γ* oscillations are a prime candidate for modulating synaptic plasticity because they reflect co-ordinated activity on the time scale of excitatory postsynaptic responses, and are within the range of relative spike timing that is optimal for spike timing dependent plasticity (10–20 ms).

Outside the motor network, it has been demonstrated that the magnitude of *γ* oscillations present at baseline accurately predicts subsequent learning across a range of tasks [54–57]. For example, in a human episodic working memory task, the degree of increase in *γ* oscillatory activity during encoding predicted subsequent recall [55]. In a rodent model, the degree of increase of activity in the *γ* band in response to a tone at baseline accurately predicted both the acquisition of subsequent associative memory, when that tone was paired with a shock, and receptive field plasticity [54].

A recent study from our group took a similar approach in the human motor system. We applied transcranial alternating current stimulation (tACS) to the M1 of healthy controls. tACS applied at 75 Hz, thought to drive activity in local *γ* circuits, led to a significant decrease in GABA_A_ activity, as assessed by TMS. Pertinently for this discussion, in a separate experimental session to the tACS application, we asked subjects to learn an explicit, visually cued serial reaction time task (SRTT), allowing us to quantify each individual’s learning ability. We demonstrated a strong relationship between 75 Hz tACS-induced change in GABA_A_ activity at rest and learning ability on a subject-by-subject basis [23•]. Taken together with the studies outside the motor domain above, this result strongly supports the hypothesis that the magnitude of *γ* oscillations are predictive of learning, and further suggests that this may be due to GABAergic activity.

As discussed above, local *γ* oscillations within a network can be coupled via long-range θ frequency activity. As learning of complex motor skills requires not only changes in local activity but also changes in communication, or functional connectivity, between regions, this raises the clear hypothesis that perhaps this θ-γ coupling may underpin at least some network-level plastic changes. In support of this rather speculative hypothesis, in vitro studies have shown that local θ-γ coupling determines whether applied stimuli result in LTP or LTD [58], and θ-γ coupling has been demonstrated to be modulated during human non-motor learning [59, 60].

## Novel Interventional Approaches to Study *γ* Oscillations

A large proportion of our understanding of neocortical *γ* oscillations has been acquired through correlational studies involving passive measurements of brain activity during a task or at rest. Recently, the development of interventional approaches, such as optogenetics in animals and tACS in humans has played a pivotal role in probing the mechanisms and neural circuits underlying neocortical *γ* oscillations and establishing for the first time a causal relationship between brain oscillations and behaviour.

Optogenetics is a powerful tool for rapid selective activation/inhibition of specific neurons and can be used to exogenously introduce oscillatory activity at a desired frequency into a specific brain region [61]. An elegant series of studies have used optogenetic manipulations in mice to show that stimulation of PV interneurons can generate LFP oscillations in vivo selectively at *γ* frequencies, whereas inhibition of PV interneurons suppresses the power of evoked oscillations at this frequency band [16, 17]. Together, these findings have provided strong evidence on a powerful role of PV interneurons in *γ* oscillations in vivo. While these studies were performed in non-motor cortical regions, the presence of inhibitory interneurons and their actions through GABA_A_ synapses are considered to be common denominators of various areas of neocortex, hippocampus, striatum and other brain regions [9].

Behavioural effects of optogenetic manipulations of *γ* oscillations have been reported in somatosensory [62] and prefrontal regions [63, 64] but very limited evidence exists in the motor domain. Constant optogenetic stimulation has been shown to elicit intrinsic propagating waves of *γ*-band (40–80 Hz) oscillations in the LFPs of the non-human primate motor cortex [2]. Interestingly, *γ* oscillations induced during movement preparation were suppressed upon voluntary movement execution, and predominantly in cortical sites distant to the stimulation (although this data included only one subject). The interpretation of these findings is difficult, particularly in light of human studies showing an increase, rather than a decrease, in M1 *γ* activity during movement execution [7, 8]. In addition, disentangling the contribution of different cell types to the measured *γ* activity is difficult as there is evidence that the optogenetic construct used is expressed primarily in pyramidal neurons and to a lesser degree in inhibitory interneurons [65], Further, recruitment of other cell types via network interactions cannot be excluded.

In humans, tACS has emerged as a promising non-invasive approach involving the application of a low amplitude alternating current of up to 2 mA (peak-to-peak) via two or more scalp electrodes. It offers the unique opportunity to drive human cortical rhythms in a frequency-specific manner and study associated behavioural performance [66, 67]. The application of *γ*-frequency (70 Hz) tACS to the left M1 has led to a frequency-specific increase in movement velocity in a visually guided movement task, thus providing first-time evidence on the causal relationship between motor cortical *γ* activity and motor behaviour [10•]. Previous studies have suggested that *γ* oscillations provide a high temporal resolution “updating system” for online motor control [68, 69], which may be vital for sudden rearrangements of the motor plan [53•]. In agreement with this, *γ* tACS has been found to improve specific components of the visuo-motor task where a sudden online rearrangement of the motor plan is required [70•]. These findings may be of clinical relevance in movement disorders, such as Parkinson’s disease, where switching from one motor program to another contributes to bradykinesia, one of the cardinal motor manifestations of the disease [71].

More recently, a novel combined tACS-functional magnetic resonance imaging (fMRI) approach has been employed to identify the neural mechanisms underlying improvements in motor performance induced by *γ* tACS over the M1 [72•]. The results have demonstrated that the degree of *γ* tACS-induced enhancements in movement performance correlates with the magnitude of the *γ* tACS-induced change in neural activity in the stimulated M1, and results in specific compensatory changes of brain activity in the remote dorsomedial prefrontal cortex. Overall, these findings suggest a direct link between the behavioural effects of motor cortical *γ* entrainment and both the modulation of local activity in the targeted site as well as compensatory modulation within connected and functionally related brain networks.

From a mechanistic point of view, another tool of an increasing value is a combined tACS-transcranial magnetic stimulation (TMS) approach, which has been utilised to assess the effects of exogenously driven oscillations on various parameters of motor excitability. As discussed previously, our recent study has demonstrated that driving motor cortical *γ* oscillations modulates GABA_A_ inhibition in the human M1, in agreement with the evidence from modelling and animal studies pointing to the key role of GABA_A_ receptor mediated inhibition in the generation of *γ* activity [9, 73]. Interestingly, *γ* tACS-induced change in inhibition was closely related to an individual’s ability to learn a motor task, suggesting for the first time that local inhibitory interneuronal circuits in M1 involved in *γ* oscillations may also be important for motor learning. In support of these findings, MRGS peak frequency, as measured using MEG, has been found to be related to performance in a motor learning task (Nowak et al., unpublished observations). This relationship was absent when the individual level of GABA_A_-mediated inhibition was controlled for.

Undoubtedly, tACS has started emerging as a valuable tool to study the mechanisms underlying cortical *γ* oscillations and their functional significance. However, a major drawback is that it is not possible to ascertain how effective the employed tACS paradigms were in driving cortical oscillations and whether, and to what extent, the exogenous stimulation changed the physical connectivity through spike timing dependent plasticity [74, 75], which in turn could have affected behaviour. To provide unequivocal evidence on tACS-induced entrainment of endogenous oscillations, concurrent MEG/EEG monitoring would be necessary [76–78], although this line of research remains a non-trivial challenge due to concerns surrounding stimulation artefact removal methods [79]. Further, the development of intermittent tACS paradigms [75, 80], with longer windows of stimulation-free periods would reduce the chance of inducing plastic changes.

## Conclusion

This review aimed to summarise the mechanisms underlying neocortical *γ* oscillations, with the primary focus on the studies performed in the motor cortex. In addition, the functional significance of motor cortical *γ* activity, including its role in plasticity was discussed. Finally, we reviewed the contribution of novel interventional approaches to our understanding of motor cortical *γ* gamma activity as well as their limitations. Addressing the limitations of these tools is likely to open a more fruitful avenue to further dissect the underlying mechanisms and the causal relationship between motor cortex *γ* activity and various aspects of motor behaviour.
